# Target‐site mutations and phenotypic failure in *Myzus persicae* against pyrethroids and carbamates in central Europe

**DOI:** 10.1002/ps.71181

**Published:** 2026-08-03

**Authors:** Jitka Stara, Jan Hubert, Pavla Pabiskova, Frantisek Kocourek

**Affiliations:** ^1^ Czech Agrifood Research Center Prague Czechia

**Keywords:** insecticide resistance, *kdr* resistance, *Myzus persicae*, resistance mutations

## Abstract

BACKGROUND

The green peach aphid, *Myzus persicae*, is a major pest and virus vector in central European agriculture. Following the neonicotinoid seed treatment ban and under climate warming, its populations have increased, raising concerns about insecticide resistance.

RESULTS

This study assessed the phenotypic resistance and underlying target‐site mutations in Czech populations of *M. persicae* from oilseed rape between 2022 and 2024. Bioassays with cypermethrin, pirimicarb, and acetamiprid revealed extreme and increasing pyrethroid resistance, whereas susceptibility to pirimicarb and acetamiprid remained high or improved compared to earlier periods. Molecular screening of 140 field‐collected aphids identified four sodium channel mutations (L1014F, M918L, M918T, L932F) and the ace2 mutation S431F. Survival under insecticide selection strongly highlighted the functional role of specific mutations: the M918L mutation was the most prevalent among cypermethrin survivors (68.75%), confirming it as the primary mechanism of pyrethroid target‐site resistance in these populations. Conversely, the S431F mutation reached complete fixation (100%) in pirimicarb survivors, serving as a definitive marker for carbamate resistance and indicating a high risk of rapid resistance development upon intensive use. Significant negative correlations between M918L and L1014F/L932F suggest divergent resistance lineages.

CONCLUSION

No clear geographical patterns were observed, indicating that resistance is driven by agricultural practices rather than regional factors. These findings highlight the rapid evolution of pyrethroid resistance and underscore the need for integrated pest management, including molecular monitoring and careful insecticide rotation, to sustain control of *M. persicae* in Czechia. © 2026 The Author(s). *Pest Management Science* published by John Wiley & Sons Ltd on behalf of Society of Chemical Industry.

## Graphical abstract

Molecular screening of 140 field‐collected *Myzus persicae* aphids revealed target‐site mutations L1014F, M918L, M918T, and L932F in the sodium channel and S431F in Ace2. The M918L mutation was the primary driver of high pyrethroid (cypermethrin) resistance, while S431F served as a definitive marker for carbamate (pirimicarb) resistance.

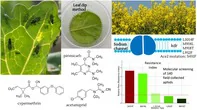

## 1 INTRODUCTION

The economic importance of *Myzus persicae* (Sulzer) lies both in its polyphagy, with a host range of over 1015 plant species worldwide, and in its role as an effective vector of plant viruses.^1^ In central Europe, it attacks a few key crops, including oilseed rape, sugar beet, potatoes, and brassica vegetables.^2^ In Czechia, *M. persicae* is the primary vector for several economically critical plant viruses, such as, Potato Virus Y (PVY) (affecting potatoes), Beet Yellows Virus (BYV) (affecting sugar beet), and Turnip Mosaic Virus (TuMV) (affecting *Brassica* crops, including oilseed rape).^3^ Overall, this species can transmit up to 180 phytopathogenic viruses.^1^ In addition to the indirect damage caused by viral infections, there is also direct damage caused by sucking, which has led to significant economic losses.^4^

Long‐term suction trap data reveal a pivotal shift in *M. persicae* populations in Czechia after 2013, with a significant increase in abundance compared to the previous decade.^5^, ^6^ This rise has amplified its role as a key virus vector in potato and other crops. The trend is attributed to a synergy of two factors: climate warming and the aphid's expansion into oilseed rape crops following the neonicotinoid seed treatment ban. The decline in protection has been most pronounced in rapeseed, where corresponding population increases have been documented across multiple European regions.^7^, ^8^

A direct measure against the spread of viruses is the application of insecticides against *M. persicae*. However, their frequent use has led to the selection of resistance in this pest to several classes of insecticides, including pyrethroids,^9^, ^10^, ^11^ carbamates,^12^, ^13^ and neonicotinoids.^14^, ^15^, ^16^ This problem is also becoming increasingly relevant in Czechia, where populations of *M. persicae* resistant to pyrethroids and carbamates have recently been reported.^17^

Pyrethroid resistance in *M. persicae* is primarily mediated by target‐site mutations in the voltage‐gated sodium channel (para‐type). Two key mutations, L1014F (kdr) and M918T (super‐kdr), confer reduced neuronal sensitivity, with the M918T mutation typically enhancing the resistance level conferred by L1014F. Notably, the M918T mutation has never been reported in the absence of the kdr (L1014F) mutation.^9^, ^18^ These mutations are widespread at high frequencies in populations across Europe, including the United Kingdom (UK), France, Spain, Italy, Germany, Belgium, and the Netherlands.^19^ Further genetic diversity has been identified with the discovery of additional mutations, such as M918L (super‐kdr‐like) and L932F.^10^, ^17^, ^20^, ^21^ In contrast to M918T, the M918L mutation can confer a resistant phenotype even in the absence of the kdr mutation (L1014F).^20^ The co‐occurrence of M918T and M918L on different alleles within a single individual has also been documented.^10^ Although the presence of resistance genes in *M. persicae* populations has been investigated in several studies,^10^, ^20^, ^21^ the effect of specific mutations on the level of aphid resistance to pesticides remains largely unknown. Some studies suggest that the M918L mutation confers greater resistance to pyrethroids than the L1014F mutation alone or in combination with the M918T mutation.^20^ However, there is insufficient knowledge of the correlation between the occurrence of mutations and the level of resistance in bioassays.

Target‐site resistance to carbamates, such as pirimicarb, in *M. persicae* is conferred by a modification of acetylcholinesterase. This mechanism is primarily driven by the S431F mutation in the ace2 gene, which causes a structural change in the enzyme, reducing its sensitivity to the insecticide.^12^, ^22^ The ace2 allele carrying the S431F mutation has been reported at high frequencies in field populations across numerous European countries, including UK, France, Spain, Italy, Belgium, and the Netherlands.^15^ This widespread prevalence underscores the significance of this resistance mechanism in field populations.

Neonicotinoid resistance in *M. persicae* is primarily mediated by two established mechanisms: the overexpression of the CYP6CY3 P450 monooxygenase and the R81T target‐site mutation. This resistance has been documented in populations across Europe, including Greece, France, and Italy.^16^, ^23^, ^24^ Recently, the spectrum of resistance mutations was expanded by the identification of a second target‐site mutation, V101I, in Chinese strains.^25^

The principal aims of this study are to: (i) quantify the prevalence of pyrethroid, carbamate and neonicotinoid resistance in Czech field populations of *M. persicae* from oilseed rape using diagnostic bioassays between 2022 and 2024; (ii) determine the spatiotemporal dynamics of this resistance across different regions of Czechia; (iii) establish the natural incidence and frequency of known resistance mutations (i.e., kdr, super‐kdr, super‐kdr‐like, MACE, and others) in these populations; (iv) evaluate the relationship between phenotypic resistance (from bioassays) and the genotypic profile of the aphids; (v) assess the frequency of resistance mutations in survivors of insecticide selection pressure during bioassays.

## 2 MATERIALS AND METHODS

### 2.1 Sample collection

A total of 28 local populations of *M. persicae* were collected from commercial oilseed rape (*Brassica napus* subsp. *napus*) crops in various regions of Czechia between September and October 2022, 2023, and 2024 (Table 1 and Fig. 1). The localities of collection represent the main regions in Czechia where winter oilseed rape is cultivated. Approximately 100 parthenogenetic female adults and nymphs were collected from oilseed rape plants in each locality. The samples were collected from commercial fields in which either no pesticides had been used or one or two applications of pyrethroids had been made, to combat *Psylliodes chrysocephala*. The aphids were collected from three sites in the field (three different areas, each approximately 24 m apart). Each local population was kept separately in a controlled environmental chamber at 20 °C, with 75% relative humidity and a photoperiod of 16 h:8 h (light/dark). The aphids were reared on kohlrabi plants (*Brassica oleracea* convar. *gongylodes*) and the parthenogenetic progeny (after three generations) were used for the bioassays.

**Table 1:** Localities in Czechia sampled for *Myzus persicae* aphids, with geographical coordinates

Year	Population number	Population	Latitude N, Longitude E (deg)
2022	1	Bezdekov	49.5778867 N, 13.8818258 E
	2	Chyst	50.1329456 N, 15.5364725 E
	3	Drouzkovice	50.4255083 N, 13.4529972 E
	4	Horazdovice	49.3339664 N, 13.6982492 E
	5	Postrekov	49.4615089 N, 12.8211331 E
	6	Risuty	50.2152347 N, 14.0176450 E
	7	Sezemice	50.0548428 N, 15.8536128 E
	8	Tasovice	48.8236111 N, 16.1275000 E
2023	9	Brno	49.1011167 N, 16.6106400 E
	10	Ceska Lipa	50.6189736 N, 14.5067622 E
	11	Louny	50.4542219 N, 13.8727489 E
	12	Prerov	49.4107011 N, 17.4763189 E
	13	Pribram	49.5566389 N, 13.8874328 E
	14	Prostejov	49.3836111 N, 17.1619439 E
	15	Zatec	50.3105561 N, 13.5625000 E
	16	Znojmo	48.8529278 N, 16.1572369 E
2024	17	Brasy	49.8499108 N, 13.5741424 E
	18	Brno	49.0361489 N, 16.6067442 E
	19	Domazlice	49.5181879 N, 12.7868970 E
	20	Ivanovice na Hane	49.2863039 N, 17.0934042 E
	21	Litomerice	50.5790673 N, 14.30828363 E
	22	Olomouc	49.5892206 N, 17.1643286 E
	23	Plzen	49.8333331 N, 13.2333331 E
	24	Prerov	49.4788222 N, 17.3676611 E
	25	Vsesulov	50.0349136 N, 13.6026508 E
	26	Uherske Hradiste	49.0419019 N, 17.3620217 E
	27	Zdar nad Sazavou	49.280724 N, 16.2273639 E
	28	Znojmo	48.8128995 N,16.18746386 E

**Figure 1:**
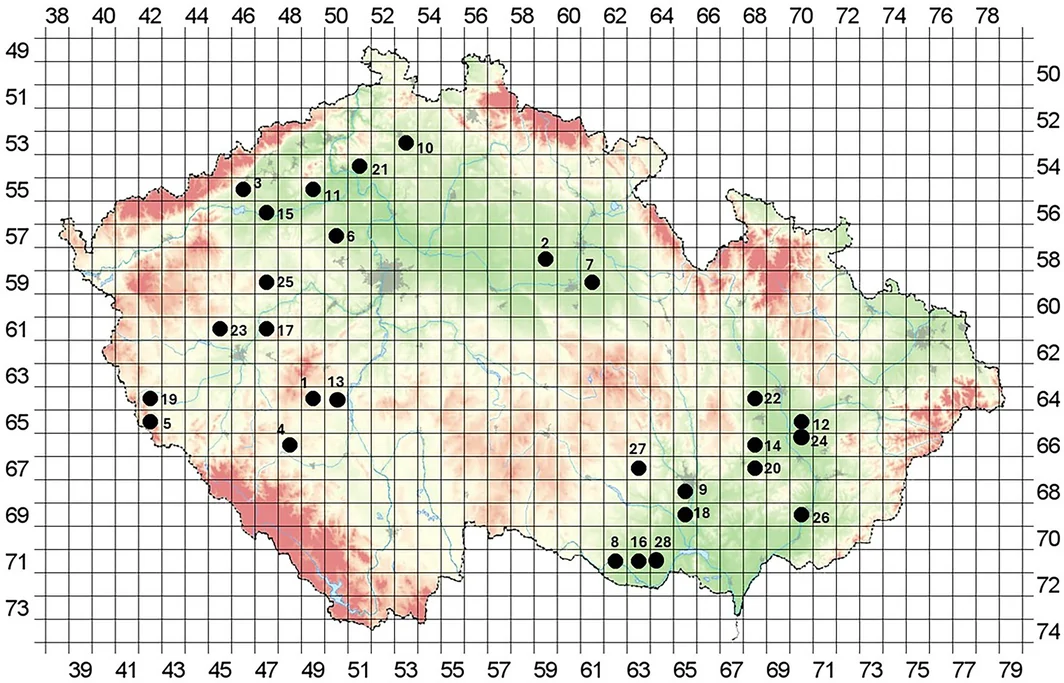
Locations selected for sampling *Myzus persicae* during 2022 and 2024. The populations corresponding to the numbers shown on the map can be found in Table 1.

### 2.2 Dose response bioassay

#### 2.2.1 Insecticides

The sensitivity of *M. persicae* populations collected in 2022–2024 was evaluated using formulated products of cypermethrin, pirimicarb and acetamiprid (Table 2). The recommended field application doses of the earlier‐mentioned insecticides were used to determine the concentrations employed in the bioassays, under the assumption that 400 L of water would be used per hectare. Four concentrations of each insecticide diluted with water were used to generate data for estimating the dose–response curves (Table 2).

**Table 2:** The recommended field rates (in bold) and tested rates of insecticides used to evaluate *Myzus persicae* susceptibility

Tested insecticides (Company)	Active ingredient (a.i.)	g a.i. L^−1^ or kg^−1^	Tested rates (mg a.i. L^−1^)
Pirimor 50 WG (Adama CZ s.r.o., Czechia)	Pirimicarb	500	25; 125; **625**; 3125
Mospilan 20 SP (Nisso Chemical Europe GmbH, Germany)	Acetamiprid	200	0.4; 4; 8; **40**
Sherpa 100 EW (SBM Developpement S.A.S.)	Cypermethrin	100	1; 5; **25**; 125

#### 2.2.2 Leaf dip method

The IRAC leaf dip test no. 019^26^ was used to evaluate the susceptibility of adult *M. persicae* to insecticides. Bioassays were conducted on *M. persicae* populations collected from oilseed rape between 2022 and 2024. Kohlrabi plant (cv. Gongylodes) leaf discs were cut, immersed in a 100 mL solution of insecticide for 10 s, dried on filter paper and placed, top side down, in plastic cups containing cooled agar (Agar Agar 700; Hages Ltd, Boskovice, Czechia), with a drop of water on the surface to help the leaf discs adhere. Using a smooth brush, 20–30 adult aphids were transferred to each leaf. The exact number of aphids transferred to each leaf was documented. Three replicate leaves per concentration were used. The cups were then sealed with breathable lids and placed in a controlled environment chamber at 20 °C with 60% relative humidity and a 16 h:8 h light/dark cycle. The laboratory efficacy of the active ingredients was expressed as aphid mortality after 72 h. The *M. persicae* alive individuals obtained from the bioassays were stored at −20 °C for further molecular analysis, which were carried out between 2023 and 2025.

### 2.3 Detection of mutations associated with resistance to pyrethroids and carbamates

Between 2023 and 2025, the total DNA of 270 *M. persicae* individuals was extracted, that is, 80 individuals that survived the field‐recommended dose (FRD) of cypermethrin (16 populations from 2022 and 2023), 50 individuals that survived FRD of pirimicarb (ten populations from 2022 and 2023), and 140 untreated individuals (28 populations from 2022–2024). Five individuals per population were analyzed separately. This sample size was chosen to enable a broad geographic survey of resistance mutation presence across multiple populations, rather than to obtain precise allele frequency estimates at the individual population level. DNA was extracted using a 10% Chelex solution. Briefly, 20 μL of deionized water was added to a test tube and the specimen was homogenized using a sterile pipette. Then, 150 μL of Chelex and 5 μL of proteinase K in concentration of 20 mg mL^−1^ were added. The specimens were boiled at 60 °C for 2 h and then at 95 °C for 15 min to inactivate the proteinase K, after which they were used as templates for polymerase chain reaction (PCR). The DNA samples were stored at −20 °C. DNA of individuals that survived FRD of cypermethrin was used to detect the M918L, M918T, L932F and L1014F mutations. DNA of individuals that survived FRD of pirimicarb was used to detect the occurrence of S431F mutation and DNA of untreated individuals was used to detect natural occurrence of all the mentioned mutations related to resistance to pyrethroids and carbamates.

DNA sequencing (Sanger method) of para gene of paratype voltage‐gated sodium channel was used to evaluate *kdr* and *super‐kdr* single nucleotide polymorphism (SNP) in one step. We sequenced PCR products from 190 *M. persicae* individuals, comprising 140 untreated field‐collected aphids (2022–2024) and 50 survivors of cypermethrin FRD bioassays (2022–2023). Fragments were amplified according to instructions for PCR using primer pairs Aph1 and Aph16.^27^

We screened for the pirimicarb resistance‐associated mutation S431F in two cohorts of *M. persicae*: 140 untreated field‐collected individuals (2022–2024) and 50 individuals that survived a pirimicarb FRD bioassay (2022–2023). Fragments 382 bp were amplified by PCR as previously described.^28^ The presence of S431F mutation was detected by restriction enzyme Sspl (Thermo Fisher Scientific, Vilnius, Lithuania). To amplify the 382 bp fragment hiding the S431F mutation, we used the allele non‐specific forward (5′MpS431F) and reverse (3′MpS431F) primers and the PCR protocol outlined by Kwon *et al*.^28^ After PCR in a C1000 Touch thermocycler (BioRad, Hercules, CA, USA), restriction was performed. The unmutated CAATATT is cleaved by the restriction endonuclease SspI AAT/ATT (Fast digest SspI restriction enzyme). The reaction product (10 μL) was added to the digestion mixture (20 μL) and incubated at 37 °C (SspI) for 5 min.^11^ The digestion mixture consisted of the restriction enzyme (0.5 unit μL^−1^) and a 1× concentration of the supplied buffer. The digestion products, as well as a ZipRuller DNA ladder express 2100 bp (Thermo Fisher Scientific, Vilnius, Lithuania) that was used as the size standard, were separated by electrophoresis on 1% agarose gels. Gels were stained in SERVA DNA Stain Clear G (Heidelberg, Germany) and imaged using a G:BOX (Syngene, Cambridge, UK). We sequenced selected individuals (ten classified as mutant and ten as wild‐type based on restriction fragment length polymorphism (RFLP) analysis). In all cases, the RFLP results were consistent with the sequencing data: individuals with the undigested fragment (382 bp) carried the S431F mutation, while those showing digested fragments carried the wild‐type allele. This validation step confirmed the reliability of the RFLP method for detecting the S431F mutation in our study.

### 2.4 Data analyses

Raw data are presented in Supporting Information, Tables S4–S7. The data from bioassays were analyzed by probit analysis using a dose‐effect analysis in R software 4.4.1.^29^ The probit model was calculated for every insecticide. The following R packages were used: ggplot2^30^ for graphical output, dplyr^31^ for data manipulation, and MASS^32^ for probit analysis and median lethal concentration (LC_50_) estimation. The insecticide concentration (LOG10) population and year and their interactions were the factors, and mortality was dependent variable (binomial distribution). The LC_50_ values, that is, concentrations required to kill 50% of populations, were fitted with a 95% confidence limit from the model. The mortality data in most cases covered a sufficient range of 5–95% at the tested concentrations. The goodness‐of‐fit of the probit model to the observed dose–response data was evaluated using Pearson's chi‐square (*χ* ^2^) test. For each population and each insecticide, we compared the observed number of dead individuals with the number expected under the fitted probit model across the tested concentration levels. In our analysis, all reported LC_50_ values (for populations where mortality at the highest tested concentration exceeded 50%) were based on models with a non‐significant Pearson's *χ* ^2^ test (*P* > 0.05). For populations where mortality at the highest tested concentration remained below 50%, LC_50_ could not be reliably estimated and these populations were excluded from quantitative LC_50_‐based comparisons, though they were included in qualitative resistance classification. For baseline populations where mortality at the lowest tested concentration exceeded 50%, LC_50_ values were estimated by extrapolation below the tested concentration range using the fitted probit model. Such extrapolation is statistically valid when the dose–response model provides a good fit for the data in the observed range.

Resistance ratios (RRs) were calculated as the LC_50_ of each population divided by the lowest LC_50_ (most susceptible baseline) for the corresponding insecticide. Due to the absence of a long‐term laboratory susceptible reference strain of *M. persicae* in Czechia, RRs were calculated using the most susceptible field population from the study period as a baseline. This approach is widely accepted and has been published in several peer‐reviewed studies.^33^, ^34^ RR served as a continuous measure for temporal trends, while categories were created as discrete variables for genotype association studies. Both approaches are complementary, providing deeper insight into resistance dynamics in *M. persicae* populations. All bioassays were conducted with the same formulated products to ensure consistency in potential formulation effects across all tested populations. The baseline LC_50_ values were: 0.38 mg L^−1^ for cypermethrin (Ceska Lipa in 2023), 0.23 mg L^−1^ for pirimicarb (Prostejov in 2023), and 0.05 mg L^−1^ for acetamiprid (Ivanovice na Hane in 2024).

*Myzus persicae* populations were classified into three resistance levels per insecticide (C1–C3, P1–P3, and A1–A3 for cypermethrin, pirimicarb, and acetamiprid, respectively) using LC_50_‐based thresholds. Group classification (C1–C3, P1–P3, A1–A3) was used for qualitative categorization to analyze relationships between phenotypic resistance and genotype (mutation presence). Discrete categories (e.g., ‘highly resistant’) allow statistical testing of mutation frequency differences among resistance levels. The classification for cypermethrin was low resistance (C1; LC_50_ 0.1–5.0 mg L^−1^), resistance (C2; LC_50_ 5.0–300.0 mg L^−1^), and high resistance (C3; LC_50_ > 50 000 mg L^−1^). The classification for pirimicarb was: high sensitivity (P1; LC_50_ less than 1.0 mg L^−1^), sensitivity (P2: LC_50_ 1.0–20.0 mg L^−1^), and low sensitivity/resistance (P3; LC_50_ more than 20.0 mg L^−1^). The classification for acetamiprid was high sensitivity (A1; LC_50_ less than 0.5 mg L^−1^), sensitivity (A2: LC_50_ 0.5–5.0 mg L^−1^), and low sensitivity (A3; LC_50_ more than 5.0 mg L^−1^). These classifications were added into the new probit models and evaluated.

Mann–Whitney analysis (included in the standard R installation) was used to compare log‐scaled RRs from populations in 2022–2024 and in 2018–2022 period (data from Stara *et al*.^17^). To analyze the relationship between RR and lethality at FRD in 2022–2024 and 2018–2022 periods, the lethality was plotted over log‐scaled RR from all populations for each pesticide. A linear model was used to fit the data as follows: *y* = *ax* − *b*, where *x* is the log‐scaled RR, *y* is the lethality at recommended active ingredient rate, *a* is the slope, and *b* is the *y*‐intercept.

Contingency tables and *χ* ^2^ analyses were used to determine the frequency of resistance mutations in *M. persicae* samples from different populations. Contingency tables and *χ* ^2^ analysis were also used to analyze the association between mutations and *M. persicae* resistance to pyrethroids and carbamates in samples from bioassays. Resistance index (RI) was calculated using the standard formula: RI = mutation frequency in pesticide group/mutation frequency in control group, where pesticide group consisted of samples from bioassays with cypermethrin or pirimicarb and control group consisted of samples without the bioassay.

Spearman correlations test was used to analyze correlation between occurrence of target‐site mutations followed by permutation test to evaluate the significance of the results.

## 3 RESULTS

### 3.1 Toxicity of insecticides to *M. persicae* populations

Efficacy of cypermethrin, pirimicarb, and acetamiprid against *M. persicae* populations varied between 2022 and 2024. Control mortality remained low (0–12.5%). For cypermethrin, mortality at the FRD was generally low and declined markedly from 2023 to 2024, with mean mortality falling to 19.22%. Sensitivity to pirimicarb was high in 2023–2024, reaching 100% mortality in most populations, while mortality did not reach 100% in any population during 2022 (range: 87.8 to < 100%). In contrast, *M. persicae* populations were consistently susceptible to acetamiprid across all years, with mortality exceeding 90% in all cases (Table 3).

**Table 3:** *Myzus persicae* mortality (%) in leaf dip bioassay in 2022–2024 at the field‐recommended dose of alpha‐cypermethrin/cypermethrin, pirimicarb and acetamiprid (three replicates per insecticide), total mean mortality (mean from all the samples), and standard deviation (SD)

Year of testing	Population	Alpha‐cypermethrin/cypermetrin	Pirimicarb	Acetamiprid
2022	Bezdekov	23.0	87.8	100
	Chyst	47.5	92.8	100
	Drouzkovice	45.4	89.5	100
	Horazdovice	60.1	93.1	95.9
	Postrekov	37.7	89.0	94.4
	Risuty	78.2	95.6	100
	Sezemice	15.9	94.2	99.3
	Tasovice	11.6	96.7	100
**Mean mortality/year (SD)**		**36.86 (23.21)**	**91.00 (5.03)**	**96.90 (5.79)**
2023	Brno	94.6	94.3	100
	Ceska Lipa	100	100	100
	Louny	61.2	100	100
	Prerov	87.6	—	100
	Pribram	49.2	100	100
	Prostejov	96	100	100
	Zatec	54.8	100	100
	Znojmo	91.2	100	100
**Mean mortality/year (SD)**		**79.33 (20.65)**	**99.19 (2.15)**	**100 (0)**
2024	Brasy	3.5	100	99
	Brno	3.6	100	100
	Domazlice	2.4	100	100
	Ivanovice na Hane	5.6	95	100
	Litomerice	1.8	91.6	100
	Olomouc	90.1	100	100
	Plzen	1.8	100	100
	Prerov	8.1	100	100
	Uherske Hradiste	1	100	100
	Vsesulov	9.8	100	99
	Zdar nad Sazavou	98.9	90.7	98.8
	Znojmo	4	100	100
**Mean mortality/year (SD)**		**19.22 (35.31)**	**98.11 (3.56)**	**99.73 (0.48)**

Lethal concentration (LC_50_) values for cypermethrin showed extreme variation (Table S1a). For ten 2024 cypermethrin populations, mortality at the highest concentration (125 mg L^−1^) was < 50%, and therefore LC_50_ values could not be calculated. These populations were classified as highly resistant (Group C3) and are presented as such in Table S1a and Fig. 2, without any numerical LC_50_ or RR estimate.

**Figure 2:**
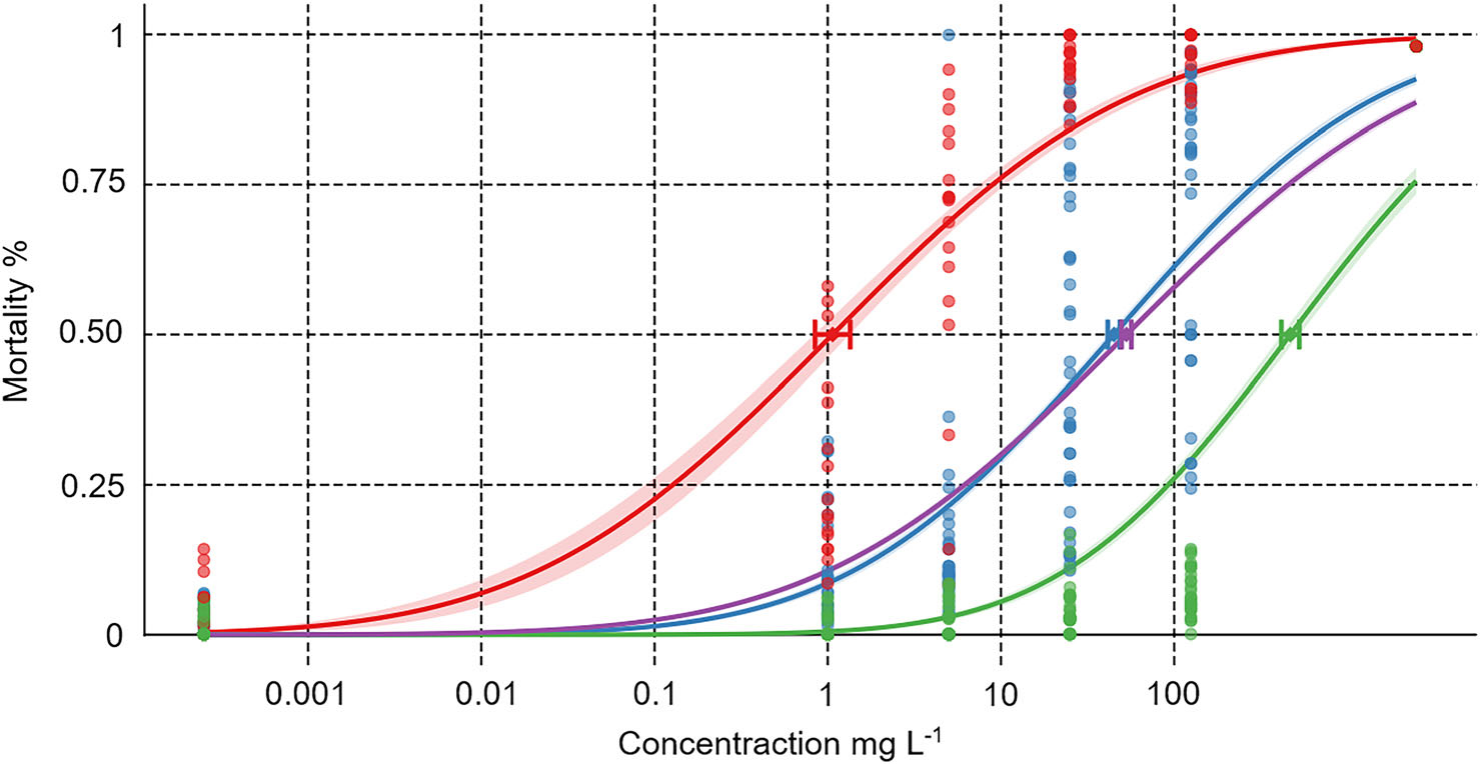
Composite log‐dose probit mortality data with 95% confidence interval of C1–C3 groups of *Myzus persicae* populations collected from different regions of Czechia in 2022–2024, related to cypermethrin (red line: C1, blue line: C2, green line: C3, violet line: original); the groups of populations C1–C3 are defined in Section 2.4.

The full model explained substantial variation in the response to cypermethrin. Population was the strongest predictor (*χ* ^2^ = 1782.8, df = 24, *P* < 0.001), followed by its interaction with log_conc (*χ* ^2^ = 571.7, df = 24, *P* < 0.001). Year (*χ* ^2^ = 353.8, df = 2, *P* < 0.001) and the year × log_conc interaction (*χ* ^2^ = 237.1, df = 2, *P* < 0.001) were also significant, while the main effect of log_conc was significant but comparatively smaller (*χ* ^2^ = 145.7, df = 1, *P* < 0.001). We acknowledge that for highly resistant populations (e.g., those with mortality at the highest concentration < 50%), future studies may benefit from exploring a broader concentration range, although such an approach was beyond the scope of the present work.

Pirimicarb LC_50_ values varied within a narrower range (0.23–86.78 mg L^−1^, Table S1b). The full model explained substantial variation in the response to pirimicarb. The strongest predictor was population (*χ* ^2^ = 1285.2, df = 24, *P* < 0.001), followed by its interaction with log_conc (*χ* ^2^ = 531.8, df = 24, *P* < 0.001). Year (*χ* ^2^ = 187.4, df = 2, *P* < 0.001) and the year × log_conc interaction (*χ* ^2^ = 71.0, df = 2, *P* < 0.001) were also significant, while the main effect of log_conc was significant but comparatively smaller (*χ* ^2^ = 463.8, df = 1, *P* < 0.001).

Acetamiprid exhibited the highest stability (0.05–8.55 mg L^−1^, Table S1c). The full model explained substantial variation in the response to acetamiprid. All predictors were statistically significant. The strongest predictors were log_conc (*χ* ^2^ = 515.86, df = 1, *P* < 0.001) and population (*χ* ^2^ = 523.02, df = 24, *P* < 0.001), which had comparable magnitudes of association. Year was also significant (*χ* ^2^ = 90.09, df = 2, *P* < 0.001). Both interaction terms were significant: year × log_conc (*χ* ^2^ = 14.97, df = 2, *P* < 0.001) and population × log_conc (*χ* ^2^ = 234.40, df = 24, *P* < 0.001). These results demonstrate that the effect of log_conc on the response is conditional upon both year and population, with the population interaction being substantially larger in magnitude.

The results of models calculated for each pesticide demonstrate that the effect of log_conc on the response is conditional upon both year and population.

Based on LC_50_ values, populations were classified into distinct groups for each compound: C1–C3, P1–P3, and A1–A3 (Tables S2a–S2c). The mean LC_50_ values defining each group spanned a wide range: from 1.07 to 469.06 mg L^−1^ for cypermethrin, 0.16 to 50.33 mg L^−1^ for pirimicarb, and 0.04 to 4.20 mg L^−1^ for acetamiprid (Table 4). The composite log‐dose probit mortality curves differed among the three susceptibility groups for each insecticide: cypermethrin (Fig. 2), pirimicarb (Fig. 3), and acetamiprid (Fig. 4).

**Table 4:** Composite log‐dose probit mortality data of groups of *Myzus persicae* populations from different regions of Czechia, related to cypermethrin, pirimicarb and acetamiprid

Pesticide	Group of populations	LC_50_ mg L^−1^	95% CL lower	95% CL upper	Slope	Intercept
Cypermethrin	C1	1.07	0.85	1.35	0.73	0.27
	C2	44.94	41.43	48.74	0.83	−1.04
	C3	469.06	416.35	528.46	0.96	−2.18
Pirimicarb	P1	0.16	0.12	0.22	0.68	0.79
	P2	4.49	3.44	5.86	0.65	−0.17
	P3	50.33	44.13	57.40	1.11	−1.45
Acetamiprid	A1	0.04	0.02	0.06	0.87	1.59
	A2	1.43	1.33	1.53	1.10	0.27
	A3	4.20	3.85	4.59	1.33	−0.30

*Note*: The median lethal concentration (LC_50_) values were estimated for groups of populations within a defined range. All reported LC_50_ values are statistically significant (*P* < 0.05) with 95% confidence limits (CLs) as shown. The groups of populations (C1–C3, P1–P3, and A1–A3) are defined in Section 2.4.

**Figure 3:**
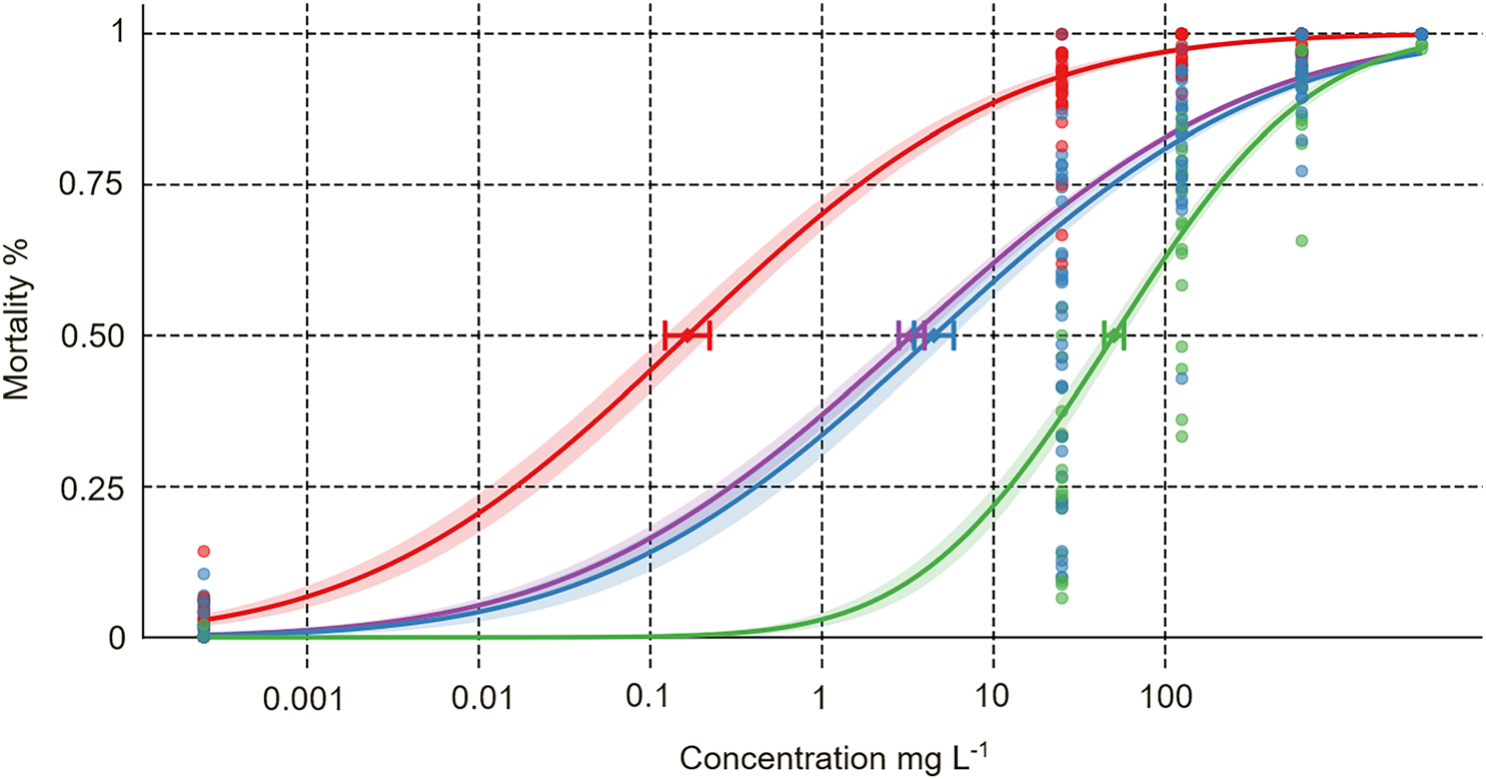
Composite log‐dose probit mortality data with 95% confidence interval of P1–P3 groups of *Myzus persicae* populations collected from different regions of Czechia in 2022–2024, related to pirimicarb (red line: P1, blue line: P2, green line: P3, violet line: original) the groups of populations P1–P3 are defined in Section 2.4.

**Figure 4:**
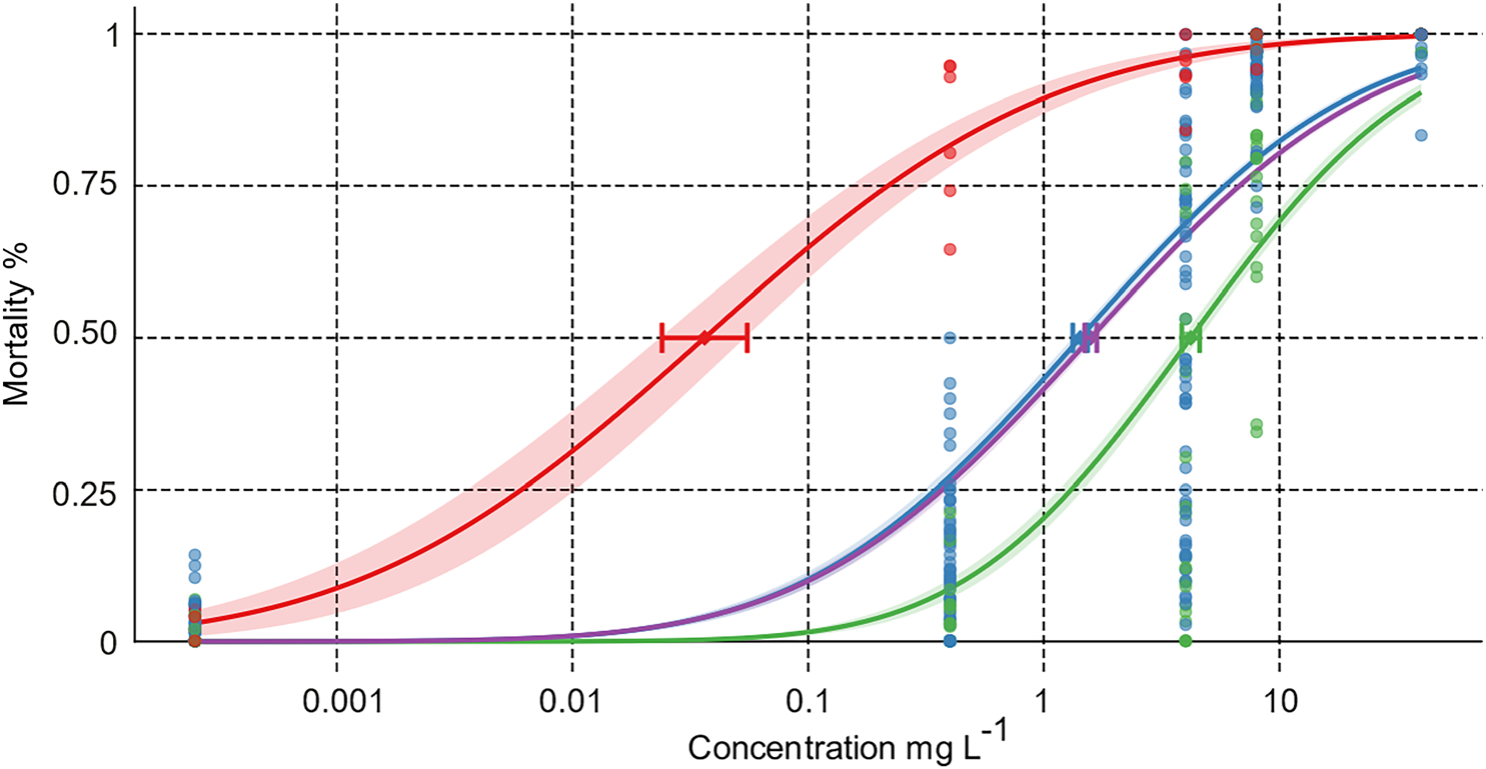
Composite log‐dose probit mortality data with 95% confidence interval of A1–A3 groups of *Myzus persicae* populations collected from different regions of Czechia in 2022–2024, related to acetamiprid (red line: A1, blue line: A2, green line: A3, violet line: original) the groups of populations A1–A3 are defined in Section 2.4.

For cypermethrin, using the FRD of 25 mg L^−1^ as a benchmark, 22% of populations exhibited low resistance (Group C1), 39% were resistant (Group C2, LC_50_ > FRD), and 39% were highly resistant (Group C3). A notable temporal pattern was observed, with the highly resistant Group C3 consisting predominantly of populations sampled in 2024, whereas Group C2 was primarily composed of populations from 2022. For pirimicarb, susceptibility was distributed as highly sensitive (37%), sensitive (41%), and low‐sensitivity/resistant (22%) populations (Table S2b). The LC_50_ values for the low‐sensitivity group (28–87 mg L^−1^) fell within an ambiguous range that precluded definitive discrimination between low inherent sensitivity and true resistance. For acetamiprid, most populations (79%) were moderately sensitive, with smaller proportions classified as highly sensitive (7%) or low‐sensitive (14%) (Table S2c). In contrast to cypermethrin, no clear temporal associations were evident for the susceptibility groups of pirimicarb or acetamiprid. Furthermore, the group distributions for all three insecticides showed no clear correlation with the regional origin of the populations (Fig. 1).

### 3.2 Temporal and spatial patterns of insecticide resistance

Resistance to cypermethrin showed extreme inter‐population variation, with RRs ranging from 2.63 (Zdar in 2024) to 203 429.82 (Tasovice in 2022) (Table S1a). No statistically significant difference was found between the mean log RR for 2018–2020 (1.96 ± 0.82 standard deviation (SD); data from Stara *et al*.^17^) and 2022–2024 (3.40 ± 2.11 SD) (*P* = 0.092). However, the higher mean in the latter period suggests a possible trend toward increased resistance, largely driven by the extreme cases from 2024.

For pirimicarb, RRs ranged from 1.52 (Plzen and Vsesulov in 2024) to 377.30 (Sezemice in 2022) (Table S1b), with historical data indicating substantially higher values in 2002 than in 2023–2024. A statistically significant decrease in resistance was observed between periods. The mean log RR was significantly higher in 2018–2020 (2.981 ± 0.841 SD; data from Stara *et al*.^17^) than in 2022–2024 (1.391 ± 0.856 SD) (*P* = 0.0003), corresponding to an approximately 39‐fold reduction in the relative risk of resistance.

Acetamiprid RRs ranged from 2.60 (Znojmo in 2024) to 139.00 (Tasovice in 2022) and were evenly distributed between 2022 and 2024 (Table S1c). Resistance levels in the recent period (2022–2024; mean log RR = 1.576 ± 0.534 SD) were substantially lower than those observed in the historical period (2018–2020; mean log RR = 2.610 ± 0.353 SD). This difference was statistically significant, as confirmed by both a Welch's *t*‐test (*P* = 0.0026) and a Mann–Whitney *U* test (*P* = 0.002). This result indicates decrease of tolerance of *M. persicae* populations to acetamiprid in 2022–2024 period.

Linear regression analysis revealed a strong and highly significant negative correlation between the cypermethrin RR (logRR) and insecticide‐induced mortality during both study periods (see Fig. 5). During the 2018–2020 period, mortality decreased sharply as resistance increased, according to the equation *y* = 111.6 − 31.5*x* (coefficient of determination (*R* ^2^) = 0.729, *P* < 0.001). A similarly strong and significant relationship was observed for the 2022–2024 period, described by the equation *y* = 89.9 − 18.9*x* (*R* ^2^ = 0.738, *P* < 0.001). A clear temporal shift was evident in the slope of the regression lines. The slope for the 2018–2020 period (−31.5) was substantially steeper than that for the 2022–2024 period (−18.9). This flattening of the dose–response relationship suggests that higher levels of resistance have become established in the *M. persicae* population, meaning that incremental increases in the RR now result in smaller reductions in field efficacy than in the earlier period.

**Figure 5:**
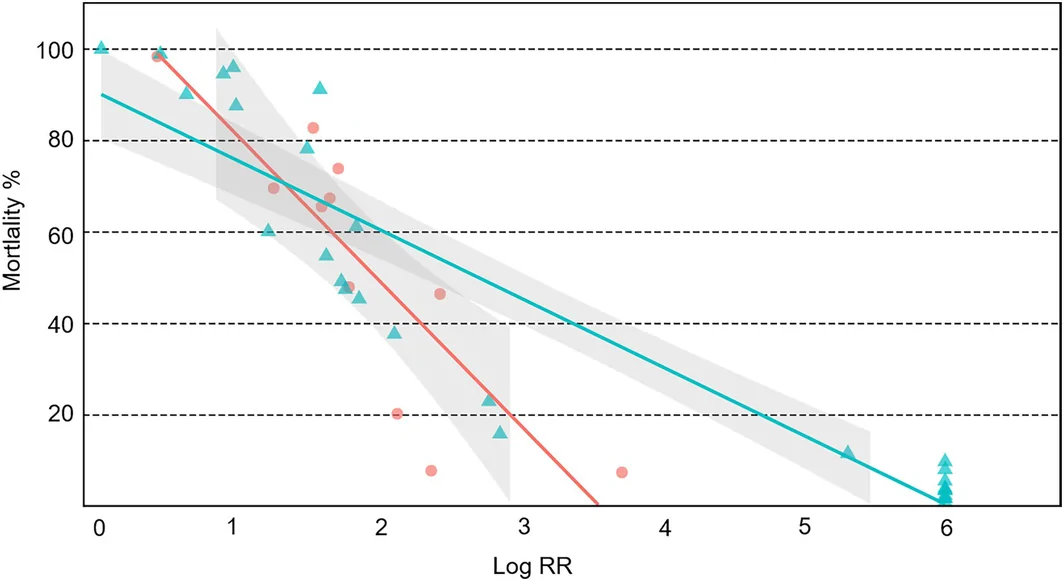
Linear regression of log RR for cypermethrin and mortality (%) of *Myzus persicae* populations in 2018–2020 period (red line; *y* = 111.6 − 31.5*x*, *R* ^2^ = 0.729 (data from *Stara et al*.^17^) and 2022–2024 (blue line; *y* = 90.1 − 14.9*x*, *R* ^2^ = 0.857). Ten populations from 2024 were excluded from the regression analysis, as no reliable RR could be calculated.

Linear regression analysis revealed a significant temporal change in the relationship between the RR (logRR) and pirimicarb‐induced mortality in *M. persicae* (see Fig. 6). For the earlier period (2018–2020), a strong and significant negative relationship was observed (*y* = 140.5 − 23.4*x*; *R* ^2^ = 0.421, *P* = 0.031). This steep slope indicates that, during this time, even moderate increases in the RR were associated with a sharp decline in mortality. In contrast, for the later period (2022–2024), the relationship remained significant, but was much weaker (*y* = 100.2 − 2.8*x*; *R* ^2^ = 0.304; *P* = 0.0029). The much shallower slope shows that the same increase in logRR now results in a much smaller reduction in mortality. Although the model is statistically significant, it explains a lower proportion of the variance in the data than in the earlier period.

**Figure 6:**
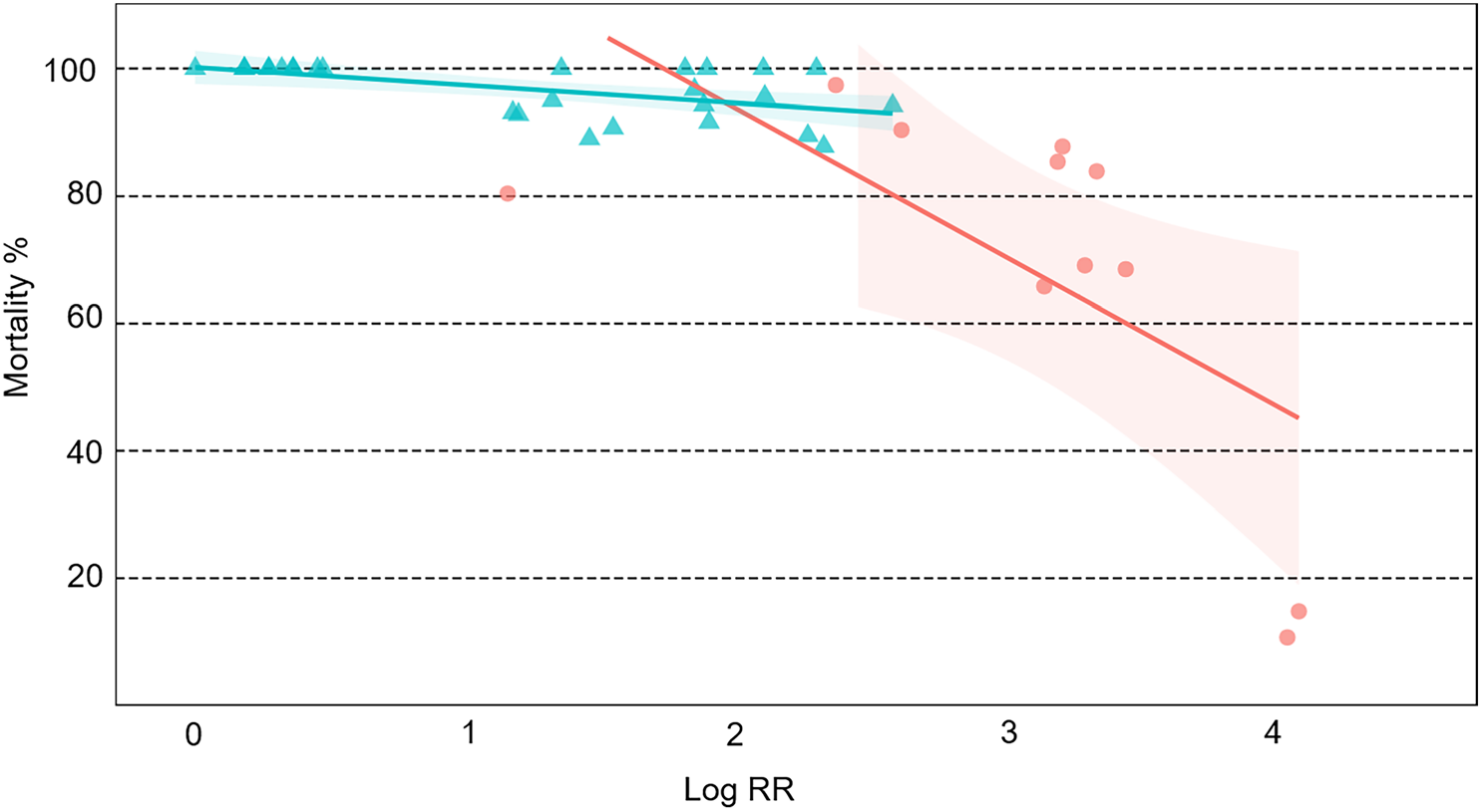
Linear regression of log RR for pirimicarb and mortality (%) of *Myzus persicae* populations in 2018–2020 period (red line; *y* = 140.5 − 23.4*x*, *R* ^2^ = 0.421 (data from Stara *et al*.^17^) and 2022–2024 (blue line; *y* = 100.2 − 2.8*x*, *R* ^2^ = 0.304).

### 3.3 Target‐site resistance mutations in field populations of *M. persicae*

Sequencing of the para sodium channel gene in 140 individual *M. persicae* from 28 field populations identified four heterozygous SNPs, each leading to an amino acid substitution associated with pyrethroid resistance: L1014F (kdr), M918L (super‐kdr‐like), M918T (super‐kdr), and L932F. The overall mutation frequencies of mutant aphids across all individuals were: M918L (55.71%), L1014F (48.57%), L932F (37.86%), and M918T (10.71%) (Table 5). All tested aphids carried at least one mutation, which occurred in four distinct genotypic patterns: M918L alone; L932F + L1014F; M918T + L1014F; and M918T + M918L + L1014F (Table S3a). Notably, the M918L substitution was the most common, followed by L1014F, indicating a widespread prevalence of these two resistance mutations in the surveyed populations.

**Table 5:** Frequency (%) of mutant aphids carrying the target‐site resistance mutations (M918T, M918L, L932F, L1014F, S431F) in *Myzus persicae* populations under natural and insecticide‐selection pressure

Population/treatment	*N*	M918T	M918L	L932F	L1014F	S431F
Natural population	140	10.71	55.71	37.86	48.57	27.14
Bioassay: cypermethrin	80	7.50	68.75	23.75	28.75	—
Bioassay: pirimicarb	80	—	—	—	—	100.00

*Note*: — = not analyzed; *N* = number of individuals.

Restriction analysis of the 140 PCR amplicons detected the S431F mutation – a carbamate‐resistance marker – in 27.14% of *M. persicae* individuals distributed across 16 populations (Tables 5 and S3a). All carriers of the S431F allele were heterozygous.

A statistically significant association was observed between the prevalence of the L932F and M918T mutations and the level of cypermethrin resistance, as defined by population groups C1–C3 (Fig. 7). The L932F mutation occurred most frequently in the intermediate resistance group C2 (*P* < 0.001). Conversely, the M918T substitution was predominantly found in the high‐resistance group C3 (*P* < 0.001). By contrast, there was no significant difference in the frequencies of the L1014F (*P* = 0.078) and M918L (*P* = 0.094) mutations among the three resistance categories. The occurrence of S431F mutation differed significantly between population groups P1–P3 with different levels of resistance to pirimicarb and was most common in group P2 (Fig. 7, *P* = 0.003).

**Figure 7:**
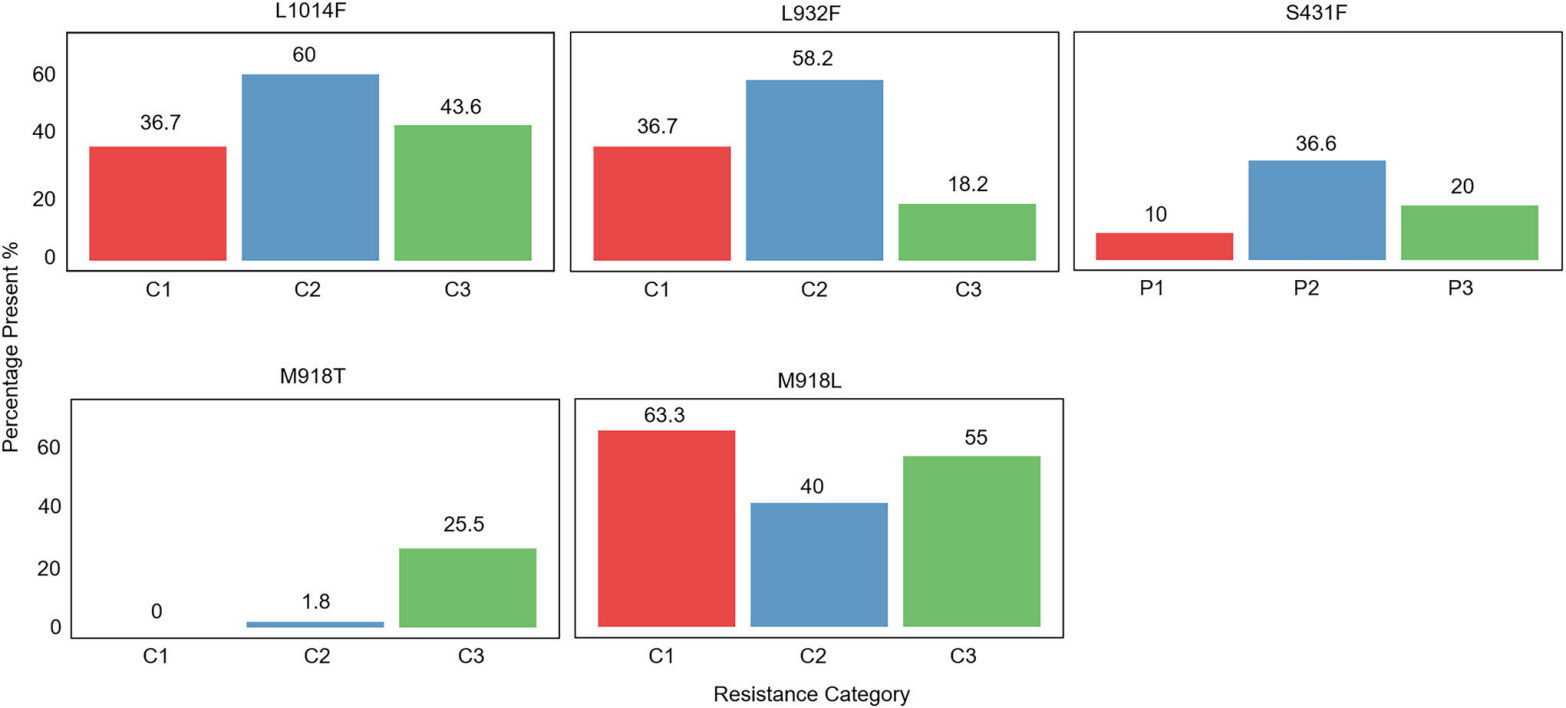
Natural occurrence of target‐site mutations M918T/L, L932F, L1014F and S431F in groups of *Myzus persicae* populations; the numbers above the columns represent the percentage of mutations that occurred, calculated from 140 individuals.

### 3.4 Correlation and linkage between target‐site mutations in field populations of *M. persicae*

Spearman correlation analysis revealed significant pairwise associations among the four resistance‐related mutations (Fig. 8). A strong positive correlation was observed between the L932F and L1014F mutations (*r*ₛ = 0.80). In contrast, strong negative correlations were found between M918L and L1014F (*r*ₛ = −0.92) as well as between M918L and L932F (*r*ₛ = −0.87). These results suggest that the M918L mutation may displace the L1014F and L932F mutations, possibly due to a higher fitness advantage in carriers. Weaker positive correlations were detected for M918T with both L1014F (*r*ₛ = 0.46) and L932F (*r*ₛ = 0.46), while the association between M918T and M918L was weakly negative (*r*ₛ = −0.35). No substantial correlation was observed between M918T and S431F (*r*ₛ = 0.20). These patterns suggest that M918L tends to occur independently of L1014F and L932F, possibly reflecting a competitive fitness relationship, whereas L932F and L1014F frequently co‐occur in the same individuals.

**Figure 8:**
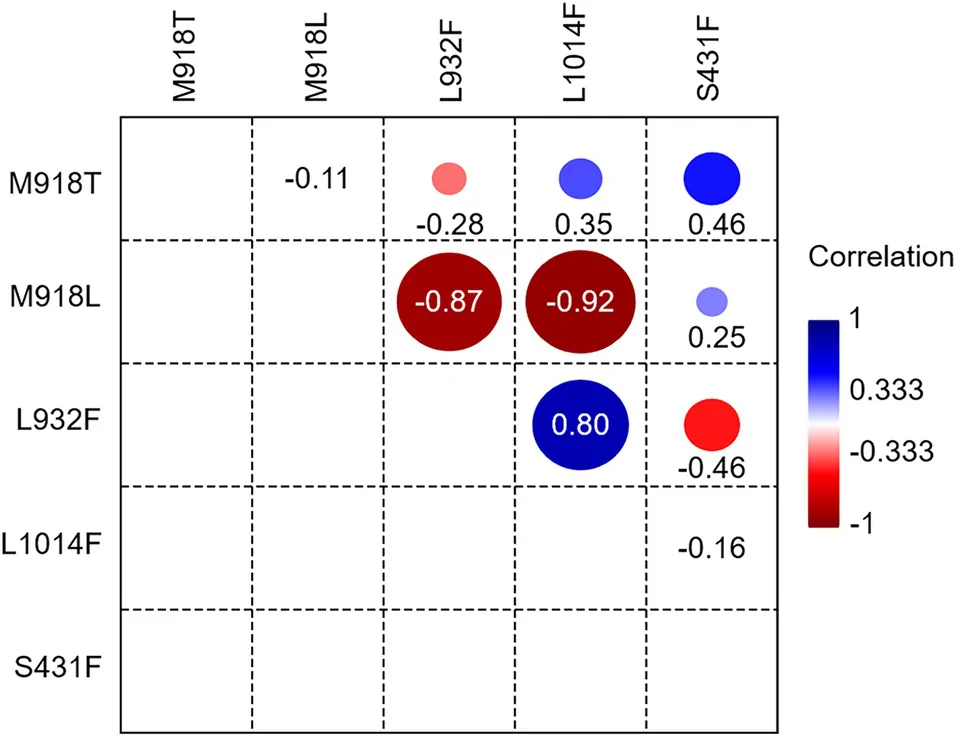
The Spearman correlation between the natural occurrence of M918T, M918L, L932F, L1014F and S431F mutations in *Myzus persicae* populations (calculated from the occurrence of mutations in 140 individuals from 28 *M. persicae* populations from 2022–2024); all shown correlations were significant in permutational test (*P* < 0.05).

### 3.5 Association of mutations with insecticide survival in *M. persicae* after laboratory selection

All the analyzed *M. persicae* individuals who survived cypermethrin FRD in the bioassay in 2022 and 2023 had one of the following mutations: M918T, M918L, L932F or L1014F. The M918L mutation was the most common occurring in 68.75% individuals. This was followed by the L1014F mutation in 28.75% individuals and the L932F mutation in 23.75% individuals. M918T occurred in only 7.5% individuals (Tables 5 and S3b).

Selection with cypermethrin in the bioassay led to change in the frequency of kdr mutations. While in the natural population the most common mutations were M918L (55.71%) and L1014F (48.57%), in individuals surviving the FRD bioassay the frequency of M918L increased to 68.75%. In contrast, the occurrence of L1014F, L932F, and M918T mutations in survivors decreased to 28.75%, 23.75%, and 7.50%, respectively. These results suggest that the M918L mutation is the main mechanism of cypermethrin target resistance in the tested populations.

The frequency of the S431F mutation differed markedly between natural populations and laboratory selection. It was present in 27.14% of field‐sampled individuals but reached complete fixation (100%) among survivors of the pirimicarb FRD bioassay (Tables 5 and S3c). This is direct evidence of strong selective pressure.

Analysis of target‐site mutations in individuals surviving the FRD of cypermethrin or pirimicarb in the 2022 and 2023 bioassays revealed a strong and specific association between the S431F mutation and resistance (Fig. 9). This mutation showed a highly significant positive association with a RI of 3.68 (*P* < 0.001).

**Figure 9:**
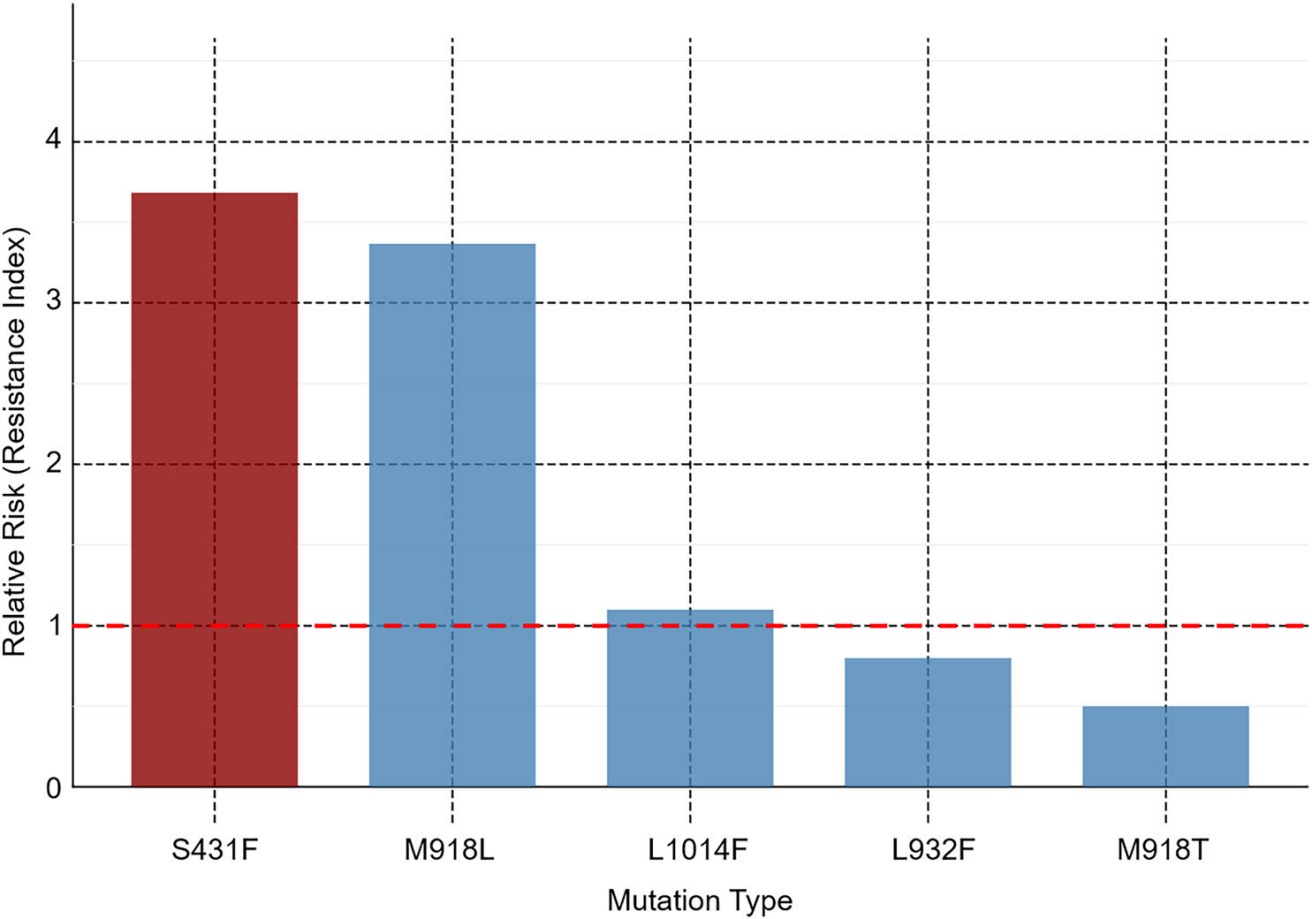
Association of target‐site mutations M918T, M918L, L932F, L1014F and S431F with *Myzus persicae* resistance to cypermethrin and pirimicarb.

The M918L mutation was also significantly associated with an increased RI (RI = 3.37, *P* < 0.001). By contrast, the L932F, L1014F and M918T mutations did not show a significant association with resistance in this assay (RI = 1.1, 0.8 and 0.5, respectively) (Fig. 9).

## 4 DISCUSSION

### 4.1 History of monitoring and controlling resistance in *M. persicae* in Czechia

Comprehensive monitoring of *M. persicae* resistance to cypermethrin (pyrethroid), pirimicarb (carbamate), and acetamiprid (neonicotinoid) has been conducted in Czechia since 2018. Following initial results from 2018–2021,^17^ this article presents findings from the second stage (2022–2024). Bioassays made it possible to evaluate trends in sensitivity to acetamiprid. This phase also involved expanding genetic screening to include the natural occurrence of target‐site mutations in relation to pyrethroids and carbamates, enabling the analysis of mutation associations.

Between 2018 and 2024, significant shifts in insecticide use influenced resistance development. Pyrethroid resistance persisted, driven by applications against spring rapeseed pests, virus vectors on potatoes and cereals, and other crops. While targeted use against *M. persicae* on winter rapeseed nearly ceased, overall application intensity of pyrethroids remained high. In contrast, carbamate selection pressure was minimal, with no use recorded between 2022 and 2024. Acetamiprid selection pressure in autumn rapeseed remained low compared to previous neonicotinoids, partly due to its limited efficacy against the main pest, *Psylliodes chrysocephala*. Low *M. persicae* prevalence in 2023–2024 further reduced applications of neonicotinoids. During 2023–2024, rapeseed protection involved flonicamid, acetamiprid, flupyradifurone (mainly as seed treatment), and pyrethroids. Spirotetramat was used on other crops until its registration ended in 2025, while diamide use (e.g., cyantraniliprole) increased significantly from 2022. The expansion of the insecticide spectrum, including substances effective against resistant populations, has been a key factor shaping *M. persicae* resistance evolution in Czechia.

### 4.2 Development of resistance of *M. persicae* to cypermethrin

Bioassay monitoring of field populations from rapeseed between 2018 and 2024 confirmed a high and widespread resistance to pyrethroids. Pyrethroid resistance in *M. persicae* persists even though targeted applications against this aphid on winter oilseed rape have nearly ceased. This is likely due to the continued intensive use of pyrethroids against other arthropod pests in the same crops, such as *Psylliodes chrysocephala* in oilseed rape, as well as in other crops like potatoes and cereals, where pyrethroids are applied against various pest species. These applications impose indirect selection pressure on *M. persicae* populations, contributing to the maintenance and further spread of resistance mechanisms even in the absence of direct targeting.

Average mortality following FRDs fell below 20% in 2023–2024. The LC_50_ value for cypermethrin showed extreme variability, exceeding 999.9 mg L^−1^ for 32% of populations. While the rate of resistance development has slowed since 2022, the number of highly resistant populations has increased. This high resistance correlates with a high frequency (69% to 8%) of four different para sodium channel mutations found in survivors after FRD application.

To document ongoing resistance evolution, a wide range of LC_50_ values for cypermethrin was recorded, indicating significant inter‐population variability. Between 2018 and 2024, LC_50_ values varied from 0.38 mg L^−1^ to > 999.99 mg L^−1^. Similarly broad variability was reported for bifenthrin.^35^ However, a key distinction emerged: while the most resistant clones in the cited study were homozygotes (R/R) for kdr and super‐kdr genotypes, our study found only heterozygous (S/R) individuals for these mutations in resistant field populations, despite comparable resistance levels.

Comparisons of resistance between geographical regions are feasible using directly comparable LC_50_ values from standardized bioassays. However, comparing published RRs is problematic due to inconsistent baseline LC_50_ values from differing ‘most sensitive’ reference populations. For instance, the LC_50_ of 0.38 mg L^−1^ identified as the most sensitive in our field study is 43 times lower than a value (16.5 mg L^−1^) reported elsewhere for pirimicarb.^28^ In this publication, RR for cypermethrin was calculated using our most sensitive field LC_50_ (0.38 mg L^−1^ from Ceska Lipa in 2023), and historical data were recalculated accordingly. This differs from Stara *et al*.,^17^ who used an LC_50_ of 0.19 mg L^−1^ from an Australian laboratory population.^36^

A limitation of this study is that for the most susceptible populations used as baselines for RR calculation, the LC_50_ values fell below the lowest tested concentration. The probit analysis allows extrapolation in such cases when mortality at the lowest concentration exceeds 50%. The future monitoring will be done to include a broader concentration range to directly estimate LC_50_ values for tested populations. The use of a field‐derived susceptible baseline rather than a laboratory reference strain may underestimate absolute resistance levels compared to studies using susceptible laboratory clones,^37^ however it still provides a valid relative comparison of resistance among populations within the region and over time.

### 4.3 Development resistance of *M. persicae* to pirimicarb

Monitoring of field populations from rapeseed between 2018 and 2024 indicates a decrease in resistance to pirimicarb. Average mortality following FRDs increased from 91% in 2020 to 98% in 2024. The LC_50_ showed low variability, ranging from 0.23 to 86.8 mg L^−1^. Since 2021, field populations have become more sensitive to pirimicarb, with high sensitivity observed in 2023 and 2024.

The detected resistance level does not fully correspond to the occurrence of the S431F mutation in local populations, found in 27% of individuals. This suggests the phenotypic expression of resistance is lower than the mutation frequency implies. However, the S431F mutation was present in all individuals surviving the FRD in bioassays. A synergistic effect between pirimicarb and piperonyl butoxide (PBO) observed in some populations between 2018 and 2020^17^ indicates that phenotypic resistance likely results from a combination of target‐site mutations and metabolic resistance. These factors may have contributed to the reappearance of sensitivity between 2020 and 2024.

In this publication, the RRs for pirimicarb were determined using the lowest LC_50_ value of 0.23 mg L^−1^ from the most sensitive field population (Prostejov in 2020–2024). Historical LC_50_ data (2018–2020) were recalculated using this same baseline. This differs from the original publication,^17^ which used an LC_50_ of 16.5 mg L^−1^ from a sensitive South Korean laboratory population.^28^

### 4.4 Sensitivity of *M. persicae* to acetamiprid

Bioassay monitoring of 28 field populations between 2018 and 2024 revealed no high‐level resistance to acetamiprid, as mortality at the FRD exceeded 90% in all populations. However, RRs showed considerable inter‐population variability, with 13 populations exhibiting RR values exceeding 50 (range: 2.60–139.00). While these RR values indicate reduced sensitivity compared to the most susceptible baseline population, they do not reach levels that would compromise field efficacy, as all populations remained highly susceptible at the FRD (mortality > 90%). This suggests that acetamiprid tolerance in Czech *M. persicae* populations is evolving, warranting continued monitoring. LC_50_ values ranged from 0.05 to 8.55 mg L^−1^.

Monitoring confirmed that reduced sensitivity to acetamiprid is a notable evolutionary trait. Even the least sensitive populations had LC_50_ values far below the FRD. This reduced sensitivity in *M. persicae* parallels that described for *Rhopalosiphum padi* to pyrethroids, observed in Australia,^38^ Ireland,^39^ and Germany.^40^ UK studies^41^ show *R. padi* populations with varying sensitivity, where the least sensitive were 32 times less sensitive than the most sensitive. Insect tolerance to insecticides often involves reduced sensitivity prior to full resistance selection. The tolerance of insects to insecticides is mostly explained by the reduced sensitivity of the pest to the active ingredient of the insecticide, or to the entire group, with the same mechanism of tolerance expression, for cases before or after resistance selection. In addition to genetic resistance, insect populations can develop reduced sensitivity (tolerance) to insecticides. This is often a metabolic‐based mechanism whereby the insect sequesters or detoxifies the active ingredient, and it is the first indication that resistance to insecticides is developing.^42^

### 4.5 Diversity and distribution of target‐site mutations

Molecular analysis has confirmed that target‐site mutations are a primary mechanism conferring resistance to pyrethroids (e.g., L1014F, M918L, M918T, L932F in the para gene) and carbamates (S431F in the ace1 gene) in European populations of *M. persicae*,^15^, ^20^ a finding now confirmed in Czech populations (Table 5). In our 2022–2024 samples from 28 field populations, we detected four distinct genotypic patterns: M918L alone; L932F + L1014F; M918T + L1014F; and M918T + M918L + L1014F. The M918L mutation was the most prevalent, consistent with its earlier dominance in 2018–2020 Czech samples.^17^ The high frequency of M918L and L1014F correlates with observed phenotypic resistance to cypermethrin, and M918L was notably the most prevalent mutation among cypermethrin survivors, confirming its central role in pyrethroid resistance. In contrast to the previous Czech study, we detected the M918T + L1014F combination for the first time in the 2022–2024 period. The analysis of five individuals per population provides a limited basis for precise estimation of allele frequencies in this study. Therefore, future studies focusing on the detailed characterization of resistance dynamics in specific populations should include larger sample sizes per site to obtain more accurate frequency estimates and better capture intra‐population genetic diversity. The methods limited to decide whether mutations such as 918T/L occur on the same DNA molecule (*cis*) or on different molecules (*trans*) remains unknown. The clonal isolation followed by Sanger sequencing will be necessary to directly resolve the haplotype configuration and fully elucidate the functional impact of these co‐occurring mutations.^9^

The prevalence of resistance mutations varies significantly across Europe. In southern and western Europe, the L1014F mutation is widespread and frequently co‐occurs with M918T, forming a haplotype linked to high‐level pyrethroid resistance in populations from Italy, Greece, Belgium, and Spain.^10^, ^11^, ^43^ A similar strong linkage exists between L1014F and L932F in regions such as France.^20^ Our data confirm these positive correlations, confirming the persistence of these linked haplotypes. In contrast, the M918L (super‐kdr‐like) mutation exhibits a distinct and often mutually exclusive pattern. First identified in the UK,^15^ it is consistently reported not to occur with L1014F.^10^, ^19^ Our findings of strong negative correlations between M918L and both L1014F and L932F support this genetic incompatibility. This suggests the evolution of at least two divergent pyrethroid‐resistance lineages in Europe: one centred on the L1014F + M918T/L932F complex and another on M918L. The strong negative correlations suggest possible competitive displacement or fitness trade‐offs, which may influence resistance dynamics under different insecticide regimes. The M918L mutation confers greater resistance to pyrethroids (types I and II) than L1014F alone or combined with M918T, potentially at a lower fitness cost.^10^, ^20^, ^35^

All field‐collected individuals carrying target‐site mutations were heterozygous (S/R), with no homozygotes detected, consistent with previous reports on *M. persicae* field populations.^21^, ^27^ Direct comparisons of resistance levels between populations with different mutation combinations should therefore be interpreted with caution, as phenotypic expression may be influenced by allelic configuration and differential gene expression.

Additionally, monoallelic expression could contribute to variable resistance phenotypes even among individuals sharing the same genotype, particularly for mutations such as M918L.^35^ Although we did not directly investigate this, the observed variability in phenotypic resistance suggests that regulatory mechanisms, including differential allelic expression, may play a role and need to be screened in the future.

Importantly, we found no clear regional pattern in the distribution of resistance in Czechia for any insecticide, suggesting that resistance is widespread and likely driven by agricultural practices rather than localized factors.

### 4.6 Variability in the evolution of target‐site resistance

The carbamate‐resistance mutation S431F shows more variable associations. Although earlier studies documented linkage between S431F and M918L,^20^, ^43^ we observed only a weak association. This discrepancy may reflect regional differences in selection pressure, host plant influence, or population genetic background. The S431F mutation also occurs independently, indicating that carbamate resistance can evolve separately from pyrethroid resistance. The S431F mutation was strongly associated with pirimicarb resistance, reaching fixation in survivors of laboratory bioassays (Table 5). This mutation serves as a clear molecular marker for carbamate resistance and its detection in over one‐quarter of field‐sampled individuals indicates a tangible risk of resistance development if pirimicarb is used intensively. The lack of temporal trends in pirimicarb and acetamiprid resistance groupings further indicates that resistance to these compounds in Czechia is not currently increasing at the population level, though vigilant monitoring remains essential.

Beyond geographic variation, the host plant on which *M. persicae* populations reside is a critical, and often deterministic, factor shaping the frequency and combination of target‐site resistance mutations. A clear dichotomy is observed between the primary woody host, peach (*Prunus persica*), and various herbaceous secondary hosts (e.g., oilseed rape, potato, sugar beet). Peach orchards, which often receive intensive insecticide treatments, act as reservoirs for the most diverse and potent resistance haplotypes. Notably, the L1014F + M918T combination, conferring high‐level pyrethroid resistance, is strongly associated with clones with holocyclic development originating from peach.^10^, ^19^ This environment appears to maintain a broad genetic pool of resistance. Our data proved the occurrence of L1014F and M918T also in *M. persicae* samples from oilseed rape.

Populations on annual crops like oilseed rape frequently exhibit a distinct resistance profile dominated by the M918L (super‐kdr‐like) mutation, which is often found in the absence of L1014F.^15^, ^20^ This pattern may result from a founder effect, where clones with the M918L genotype successfully colonized this crop system and proliferated, coupled with crop‐specific insecticide selection pressures that differ from those in orchards. The strong negative correlations we observed between M918L and the peach‐associated mutations (L1014F, L932F) further underscore this host‐based genetic divergence.

The evolution of resistance in aphid is modulated by the life cycle.^14^ *Myzus persicae* can undergo both sexual (holocyclic) and asexual (anholocyclic) reproduction.^44^ In temperate regions, the holocycle of *M. persicae*, involving sexual reproduction and overwintering of eggs, occurs alongside a mixture of holocyclic and anholocyclic clones. The existence of sexual reproduction increases diversity and allows the appearance of new combinations of mutations, while parthenogenesis can allow the rapid multiplication of a huge number of individuals carrying the selected combinations of mutations. *Myzus persicae* populations in Czechia exhibit both holocyclic and anholocyclic life cycles. The proportions of each type vary from year to year, depending on weather conditions. Holocyclic populations overwinter as eggs on primary hosts such as *Prunus persica*, while anholocyclic populations overwinter parthenogenetically on secondary hosts such as oilseed rape.

Interestingly, the S431F mutation itself does not show a strong, exclusive association with any particular host plant.^19^ Its presence across peach, oilseed rape, and other crops indicates that selection for carbamate resistance is widespread and can evolve independently within populations associated with different host plants, although *M. persicae* remains a highly polyphagous species capable of host alternation. This broad distribution underscores the pervasive historical use of carbamate insecticides.

Phenotypic expression of resistance in *M. persicae* may be influenced by mutations at the target site or metabolic resistance.^15^ Phenotypic resistance to acetamiprid may be preceded by the gradual development of tolerance. As our research shows, resistance to acetamiprid has not been observed in the phenotype after selection pressure induced by neonicotinoids since 2018. In the evolution of resistance in *M. persicae*, the expression of resistance in the phenotype may be influenced by other mechanisms. Panini *et al*.^35^ revealed a mechanism of insecticide resistance resulting from transposon‐mediated insertion mutagenesis of a genetically dominant insecticide‐sensitive allele, which allows the activation of previously unavailable recessive resistance alleles.

### 4.7 Conclusions and management implications

This study documents a concerning escalation in pyrethroid resistance among *M. persicae* populations in Czechia. Our results showed that the S431F and M918L mutations significantly increase the level of resistance in the phenotype after pyrethroid selection. No resistance to acetamiprid was observed, and susceptibility to pirimicarb has improved in recent years, likely due to reduced field use. The absence of clear geographical patterns indicates that resistance evolution is primarily driven by agricultural practices rather than regional factors. These findings underscore the urgent need for integrated pest management strategies that incorporate molecular monitoring, insecticide rotation, and the promotion of non‐chemical controls to mitigate resistance development and preserve the efficacy of existing insecticides. Future work should focus on understanding the fitness costs of resistance mutations and evaluating the impact of insecticide use patterns on resistance selection in the field.

## FUNDING INFORMATION

This research was supported by the Ministry of Agriculture of the Czech Republic grant number QL24010167.

## Supporting information


**Table S1a.** Composite log‐dose probit mortality of *Myzus persicae* collected from different regions of Czechia for cypermethrin. The resistance level was evaluated using resistance ratio (RR). All reported LC_50_ values are statistically significant (*P* < 0.05) with 95% confidence limits as shown.
**Table S1b.** Composite log‐dose probit mortality of *Myzus persicae* collected from different regions of Czechia for pirimicarb. The resistance level was evaluated using resistance ratio (RR). All reported LC_50_ values are statistically significant (*P* < 0.05) with 95% confidence limits as shown.
**Table S1c.** Composite log‐dose probit mortality of *Myzus persicae* collected from different regions of Czechia for acetamiprid. The resistance level was evaluated using resistance ratio (RR). All reported LC_50_ values are statistically significant (*P* < 0.05) with 95% confidence limits as shown.
**Table S2a.** Groups of *Myzus persicae* populations according to LC_50_ values for cypermethrin.
**Table S2b.** Groups of *Myzus persicae* populations according to LC_50_ values for pirimicarb.
**Table S2c.** Groups of *Myzus persicae* populations according to LC_50_ values for acetamiprid.
**Table S3a.** Natural occurrence of M918T/L, L932F, L1014F and S431F mutations in *Myzus persicae* populations.
**Table S3b.** Occurrence of M918T/L, L932F and L1014F mutations in *Myzus persicae* individuals who survived the FRD of cypermethrin in the bioassay.
**Table S3c.** Occurrence of S431F mutation in *Myzus persicae* individuals who survived the FRD of pirimicarb in the bioassay.
**Table S4.** Experimental data on pesticide effects across different locations.
**Table S5.** Dataset of cypermethrin field bioassays (2022–2024) stratified by experimental group (C1–C3), including application rates (g ha^−1^) and mortality counts (*N*, Nm) across locations.
**Table S6.** Dataset of acetamiprid field bioassays (2022–2024) stratified by experimental group (A1–A3), including application rates (g ha^−1^) and mortality counts (*N*, Nm) across locations.
**Table S7.** Dataset of pirimicarb field bioassays (2022–2024) stratified by experimental group (P1–P3), including application rates (g ha^−1^) and mortality counts (*N*, Nm) across locations.

## Data Availability

The data that supports the findings of this study are available in the supplementary material of this article.

## References

[1] Blackman RL and Eastop VF , Aphids on the World's Trees: An Identification and Information Guide, 1st edn. CAB International, Wallingford (1994). 10.1079/9780851988771.0000.

[2] Blackman RL and Eastop VF , Aphids on the World's Crops: An Identification and Information Guide, 2nd edn. John Wiley & Sons, Chichester (2000).

[3] Kreuze JF , Souza‐Dias JAC , Jeevalatha A , Figueira AR , Valkonen JPT and Jones RAC , Viral diseases in potato: its agricultural, nutritional and social contribution to humankind, in The Potato Crop, ed. by Campos H and Ortiz O . Springer, Cham, pp. 389–430 (2020). 10.1007/978-3-030-28683-5_11.

[4] Ali J , The Peach Potato Aphid (Myzus persicae): Ecology and Management, 1st edn. CRC Press, Boca Raton, FL (2023). 10.1201/9781003400974.

[5] Slavikova L , Fryc D and Kumar Kundu J , Analysis of twenty years of suction trap data on the flight activity of *Myzus persicae* and *Brevicoryne brassicae*, two main vectors of oilseed rape infection viruses. Agronomy 14:1931 (2024). 10.3390/agronomy14091931.

[6] Kocourek F , Douda O , Stara J , Rychly S , Fryc D , Dolezal P *et al*., Modelling flight activity of aphids in seed potatoes using suction trap and yellow water trap for risk assessment of virus diseases. Agronomy 15:1656 (2025). 10.3390/agronomy15071656.

[7] Dewar AM , The adverse impact of the neonicotinoid seed treatment ban on crop protection in oilseed rape in the United Kingdom. Pest Manag Sci 73:1305–1309 (2017). 10.1002/ps.4511. 28019077

[8] Luquet M , Poggi S , Buchard C , Plantegenest M and Tricault Y , Predicting the seasonal flight activity of *Myzus persicae*, the main aphid vector of virus yellows in sugar beet. Pest Manag Sci 79:4508–4520 (2023). 10.1002/ps.7653. 37421357

[9] Martinez‐Torres D , Foster SP , Field LM , Devonshire AL and Williamson MS , A sodium channel point mutation is associated with resistance to DDT and pyrethroid insecticides in the peach‐potato aphid, *Myzus persicae* (Sulzer) (Hemiptera: Aphididae). Insect Mol Biol 8:339–346 (1999). 10.1046/j.1365-2583.1999.83121.x. 10469251

[10] Panini M , Anaclerio M , Puggioni V , Stagnati L , Nauen R and Mazzoni E , Presence and impact of allelic variations of two alternative *s‐kdr* mutations, M918T and M918L, in the voltage‐gated sodium channel of the green peach aphid *Myzus persicae* . Pest Manag Sci 71:878–884 (2015). 10.1002/ps.3927. 25346186

[11] Margaritopoulos JT , Kati AN , Voudouris CC , Skouras PJ and Tsitsipis JA , Long‐term studies on the evolution of resistance of *Myzus persicae* (Hemiptera: Aphididae) to insecticides in Greece. Bull Entomol Res 111:1–16 (2021). 10.1017/S0007485320000334. 32539892

[12] Moores GD , Devine GJ and Devonshire AL , Insecticide‐insensitive acetylcholinesterase can enhance esterase‐based resistance in *Myzus persicae* and *Myzus nicotianae* . Pestic Biochem Physiol 49:114–120 (1994). 10.1006/pest.1994.1038.

[13] Cassanelli S , Cerchiari B , Giannini S , Bizzaro D , Mazzoni E and Manicardi GC , Use of the RFLP‐PCR diagnostic test for characterizing MACE and *kdr* insecticide resistance in the peach potato aphid *Myzus persicae* . Pest Manag Sci 61:91–96 (2005). 10.1002/ps.954. 15593078

[14] Margaritopoulos JT , Skouras PJ , Nikolaidou P , Manolikaki J , Maritsa K , Tsamandani K *et al*., Insecticide resistance status of *Myzus persicae* (Hemiptera: Aphididae) populations from peach and tobacco in mainland Greece. Pest Manag Sci 63:821–829 (2007). 10.1002/ps.1409. 17573686

[15] Bass C , Puinean AM , Zimmer CT , Denholm I , Field LM , Foster SP *et al*., The evolution of insecticide resistance in the peach potato aphid, Myzus persicae. Insect Biochem Mol Biol 51:41–51 (2014). 10.1016/j.ibmb.2014.05.003. 24855024

[16] Panini M , Dradi D , Marani G , Butturini A and Mazzoni E , Detecting the presence of target‐site resistance to neonicotinoids and pyrethroids in Italian populations of *Myzus persicae* . Pest Manag Sci 70:931–938 (2014). 10.1002/ps.3630. 23963887

[17] Stara J , Hovorka T , Horska T , Zuskova E and Kocourek F , Pyrethroid and carbamate resistance in Czech populations of *Myzus persicae* (Sulzer) from oilseed rape. Pest Manag Sci 80:2342–2352 (2024). 10.1002/ps.7646. 37402271

[18] Eleftherianos I , Foster SP , Williamson MS and Denholm I , Characterization of the M918T sodium channel gene mutation associated with strong resistance to pyrethroid insecticides in the peach‐potato aphid, *Myzus persicae* (Sulzer). Bull Entomol Res 98:183–191 (2008). 10.1017/S0007485307005524. 18076786

[19] Singh KS , Cordeiro EMG , Troczka BJ , Pym A , Mackisack J , Mathers TC *et al*., Global patterns in genomic diversity underpinning the evolution of insecticide resistance in the aphid crop pest *Myzus persicae* . Commun Biol 4:847 (2021). 10.1038/s42003-021-02373-x. PMC8263593 34234279

[20] Fontaine S , Caddoux L , Brazier C , Bertho C , Bertolla P , Micoud A *et al*., Uncommon associations in target resistance among French populations of *Myzus persicae* from oilseed rape crops. Pest Manag Sci 67:881–885 (2011). 10.1002/ps.2224. 21710686

[21] Hlaoui A , Chiesa O , Figueroa CC , Souissi R , Mazzoni E and Boukhris‐Bouhachem S , Target site mutations underlying insecticide resistance in Tunisian populations of *Myzus persicae* (Sulzer) on peach orchards and potato crops. Pest Manag Sci 78:1594–1604 (2022). 10.1002/ps.6778. 34984812

[22] Nabeshima T , Kozaki T , Tomita T and Kono Y , An amino acid substitution on the second acetylcholinesterase in the pirimicarb‐resistant strains of the peach potato aphid, Myzus persicae. Biochem Biophys Res Commun 307:15–22 (2003). 10.1016/S0006-291X(03)01101-X. 12849975

[23] Philippou D , Field L and Moores G , Metabolic enzyme(s) confer imidacloprid resistance in a clone of *Myzus persicae* (Sulzer) (Hemiptera: Aphididae) from Greece. Pest Manag Sci 66:390–395 (2010). 10.1002/ps.1888. 19950404

[24] Slater R , Paul VL , Andrews M , Garbay M and Camblin P , Identifying the presence of neonicotinoidresistant peach‐potato aphid (*Myzus persicae*) in the peach‐growing regions of southern France and northern Spain. Pest Manag Sci 68:634–638 (2012). 10.1002/ps.2307. 22052736

[25] Xu X , Ding Q , Wang X , Wang R , Ullah F , Gao X *et al*., V101I and R81T mutations in the nicotinic acetylcholine receptor β1 subunit are associated with neonicotinoid resistance in *Myzus persicae* . Pest Manag Sci 78:1500–1507 (2022). 10.1002/ps.6768. 34962090

[26] IRAC (Insecticide Resistance Action Committee) , The IRAC Mode of Action Classification Online (2022). https://irac-online.org/modes-of-action/ accessed 17 December 2025.

[27] Anstead JA , Williamson MS , Eleftherianos I and Denholm I , High‐throughput detection of knockdown resistance in *Myzus persicae* using allelic discriminating quantitative PCR. Insect Biochem Mol Biol 34:871–877 (2004). 10.1016/j.ibmb.2004.06.002. 15262290

[28] Kwon DH , Choi BR , Lee SW , Clark JM and Lee SH , Characterization of carboxylesterase‐mediated pirimicarb resistance in *Myzus persicae* . Pestic Biochem Physiol 93:120–126 (2009). 10.1016/j.pestbp.2008.12.001.

[29] R Development Core Team , R: A Language and Environment for Statistical Computing, Version 4.4.1. R Foundation for Statistical Computing, Vienna (2024) http://www.R-project.org accessed 10 March 2024.

[30] Wickham H , ggplot2: Elegant Graphics for Data Analysis, 2nd edn. Springer, Cham (2016). 10.1007/978-3-319-24277-4.

[31] Wickham H , Francois R , Henry L , Muller K , Vaughan D and Posit Software , dplyr: A Grammar of Data Manipulation. CRAN ‐ The Comprehensive R Archive Network, Vienna, Austria, (2026) https://cran.r-project.org/web/packages/dplyr/ accessed 12 May 2026).

[32] Ripley B , Venables B , Bates DM , Hornik K , Gebhardt A and Firth D , MASS: Support Functions and Datasets for Venables and Ripley's MASS. CRAN ‐ The Comprehensive R Archive Network, Vienna, Austria (2025) https://cran.r-project.org/web/packages/MASS/ accessed 12 May 2026).

[33] Pietrantonio PV , Junek TA , Parker R , Mott D , Siders K , Troxclair N *et al*., Detection and evolution of resistance to the pyrethroid cypermethrin in *Helicoverpa zea* (Lepidoptera: Noctuidae) populations in Texas. Environ Entomol 36:1174–1188 (2007). 10.1603/0046-225X(2007)36[1174:DAEORT]2.0.CO;2. 18284743

[34] Montezano DG , Hunt TE , Souza D , Vieira BC , Velez AM , Kruger GR *et al*., Bifenthrin baseline susceptibility and evaluation of simulated aerial applications in *Striacosta albicosta* (Lepidoptera: Noctuidae). J Econ Entomol 112:2915–2922 (2019). 10.1093/jee/toz237. PMC6900747 31504657

[35] Panini M , Chiesa O , Troczka BJ , Mallot M , Manicardi GC , Cassanelli S *et al*., Transposon‐mediated insertional mutagenesis unmasks recessive insecticide resistance in the aphid *Myzus persicae* . Proc Natl Acad Sci U S A 118:e2100559118 (2021). 10.1073/pnas.2100559118. PMC8201860 34074777

[36] Umina PA , Edwards O , Carson P , Van Rooyen A and Anderson A , High levels of resistance to carbamate and pyrethroid chemicals widespread in Australian *Myzus persicae* (Hemiptera: Aphididae) populations. J Econ Entomol 107:1626–1638 (2014). 10.1603/EC14063. 25195456

[37] Nauen R and Denholm I , Resistance of insect pests to neonicotinoid insecticides: current status and future prospects. Arch Insect Biochem Physiol 58:200–215 (2005). 10.1002/arch.20043. 15756698

[38] Umina PA , Reidy‐Crofts J , Babineau M , Maino JL and Edwards OR , Susceptibility of the bird cherry‐oat aphid, *Rhopalosiphum padi* (Hemiptera: Aphididae), to four insecticides. Austral Entomol 59:838–844 (2020). 10.1111/aen.12490.

[39] Walsh L , Ferrari E , Foster S and Gaffney MT , Evidence of pyrethroid tolerance in the bird cherry‐oat aphid *Rhopalosiphum padi* in Ireland. Outlooks Pest Manag 31:5–9 (2020). 10.1564/v31_feb_02.

[40] Leybourne DJ , Melloh P and Martin EA , Common facultative endosymbionts do not influence sensitivity of cereal aphids to pyrethroids. Agric Forest Entomol 25:344–354 (2023). 10.1111/afe.12539.

[41] Leybourne DJ , Baird E , Coston D , Jones R and Musa N , Molecular screening and dose‐response assays provide insights into pyrethroid resistance in four agriculturally important aphid species. Crop Prot 197:107315 (2025). 10.1016/j.cropro.2025.107315.

[42] Bass C and Nauen R , The molecular mechanisms of insecticide resistance in aphid crop pests. Insect Biochem Mol Biol 156:103937 (2023). 10.1016/j.ibmb.2023.103937. 37023831

[43] Mingeot D , Hautier L and Jansen J‐P , Structuration of multilocus genotypes associated with insecticide resistance of the peach potato aphid, *Myzus persicae* (Sulzer), in potato fields in southern Belgium. Pest Manag Sci 77:482–491 (2021). 10.1002/ps.6045. 32812675

[44] Blackman RL , Life‐cycle variation of *Myzus persicae* (Sulz.) (Hom., Aphididae) in different parts of the world, in relation to genotype and environment. Bull Entomol Res 63:595–607 (1974). 10.1017/S0007485300047830.

